# Functionalization of Tailored Porous Carbon Monolith for Decontamination of Radioactive Substances

**DOI:** 10.3390/ijms23095116

**Published:** 2022-05-04

**Authors:** Joonwon Bae, Gyo Eun Gu, Yeon Ju Kwon, Jea Uk Lee, Jin-Yong Hong

**Affiliations:** 1 Department of Applied Chemistry, Dongduk Women’s University, Seoul 02748, Korea; redsox7@dongduk.ac.kr; 2 Center for C1 Gas & Carbon Convergent Research, Korea Research Institute of Chemical Technology (KRICT), 141 Gajeong-ro, Yuseong-gu, Daejeon 34114, Korea; goo9@aekyung.com (G.E.G.); kyj0905@krict.re.kr (Y.J.K.); 3 Department of Advanced Materials Engineering for Information and Electronics, Integrated Education Institute for Frontier Science & Technology (BK21 Four), Kyung Hee University, 1732 Deogyeong-daero, Giheung-gu, Yongin-si 17104, Korea

**Keywords:** exfoliated graphene, porous carbon, functionalization, radionuclide, decontamination

## Abstract

As the control over radioactive species becomes critical for the contemporary human life, the development of functional materials for decontamination of radioactive substances has also become important. In this work, a three-dimensional (3D) porous carbon monolith functionalized with Prussian blue particles was prepared through removal of colloidal silica particles from exfoliated graphene/silica composite precursors. The colloidal silica particles with a narrow size distribution were used to act a role of hard template and provide a sufficient surface area that could accommodate potentially hazardous radioactive substances by adsorption. The unique surface and pore structure of the functionalized porous carbon monolith was examined using electron microscopy and energy-dispersive X-ray analysis (EDS). The effective incorporation of PB nanoparticles was confirmed using diverse instrumentations such as X-ray diffraction (XRD), Fourier-transform infrared (FT-IR), and X-ray photoelectron spectroscopy (XPS). A nitrogen adsorption/desorption study showed that surface area and pore volume increased significantly compared with the starting precursor. Adsorption tests were performed with ^133^Cs ions to examine adsorption isotherms using both Langmuir and Freundlich isotherms. In addition, adsorption kinetics were also investigated and parameters were calculated. The functionalized porous carbon monolith showed a relatively higher adsorption capacity than that of pristine porous carbon monolith and the bulk PB to most radioactive ions such as ^133^Cs, ^85^Rb, ^138^Ba, ^88^Sr, ^140^Ce, and ^205^Tl. This material can be used for decontamination in expanded application fields.

## 1. Introduction

Recently, the need for intensive energy sources has increased dramatically, because human life and activities are becoming complex and energy-consuming. At the same time, a significant interest has been drawn to nuclear energy and technology dealing with dangerous radioactive substances to increase energy production capacity/efficiency, leading to a rapid increase in nuclear waste production. Therefore, it becomes even more important for us to control the potentially hazardous nuclear wastes effectively. Even if several methods have been developed to remove the nuclear wastes, it is still a challenging task to remove the radioactive substances or produce functional media materials for contamination control [1,2,3,4,5,6].

Since the Fukushima accidents, a desire for the development of environmentally benign and functional materials for decontamination of radioactive substances is ever increasing. Among various strategies to remove the radioactive species, adsorption with porous materials can be promising, because of easy preparation and simple use [7,8,9,10,11,12]. It is known that the porous materials can be manufactured easily from various precursor materials and accommodate a large amount of substances due to high surface area and pore volume [13,14,15,16,17,18]. To date, numerous porous materials have been produced using diverse methods, such as the template approach, porogen addition, and self-assembly [19,20]. The selection of precursor and methodology has become wider and the resulting porous materials can be customized depending on their usage and purpose, for example, storage and release of chemical species such as energy, gas molecules, and drugs [21,22,23,24,25,26,27].

In this study, a functional porous carbon monolith (PCM) decorated with the Prussian blue (PB) nanoparticles (PB@PCM) was produced for removal of the radioactive substances by selective removal of colloidal silica microparticles working as the hard template and porogen from exfoliated graphene/silica composite precursors, which was easily obtained by direct mixing of graphene and colloidal silica particles. This approach was simple because the preparation and removal of the hard template with a complicated and tailored structure could be avoided. In addition, it was expected that an unprecedented pore structure could be generated because graphene/silica composite precursors possessing an irregular pore structure were introduced. An interconnected 3D pore structure with an increased surface area was generated after removal of the colloidal silica particles, as a controlled pore structure with a similar pore size was not mandatory for this study. However, it was important to analyze the pore structure and surface properties to see if the obtained material could be used for the decontamination of radioactive species. As the removal of radioactive materials was strongly dependent on the adsorption mechanism, a high surface area of the PCM was one of the most critical prerequisites. Moreover, the surface properties and pore structure were analyzed extensively using various instrumentations.

On the other hand, the adsorption of radioactive species was promoted by the addition of PB nanoparticles to the internal structure of the PCM material, because it was reported that the PB, known as ferric(III) hexacyanoferrate(II), has been the most effective adsorptive material for cesium (Cs^+^) ions. [28,29,30,31,32]. In particular, the cage size of PB crystal is similar to the hydration radius of a cesium ion, resulting in excellent binding ability and selectivity of the Cs^+^ ions [33,34,35,36,37,38,39]. Due to the unique internal pore structure and increased surface area of the PCM material, the incorporation of the PB nanoparticles into the PCM materials could be promoted. Subsequently, adsorption tests were conducted with a typical radioactive ion, ^133^Cs, to observe adsorption isotherms using both Langmuir and Freundlich isotherms and calculate kinetic parameters associated with the adsorption behavior. The PB-decorated PCM showed a higher adsorption capacity to typical radioactive ions ^133^Cs, ^85^Rb, ^138^Ba, ^88^Sr, ^140^Ce, and ^205^Tl than the precursors and the bulk PB. The newly developed material and method might offer an opportunity for future research and investigation of nuclear waste control. Moreover, it can be transplanted to diverse technologies for waste removal and treatment.

## 2. Results and Discussion

### 2.1. Preparation of the Porous Carbon Monolith with Tailored Pore Structure

**Figure 1:**
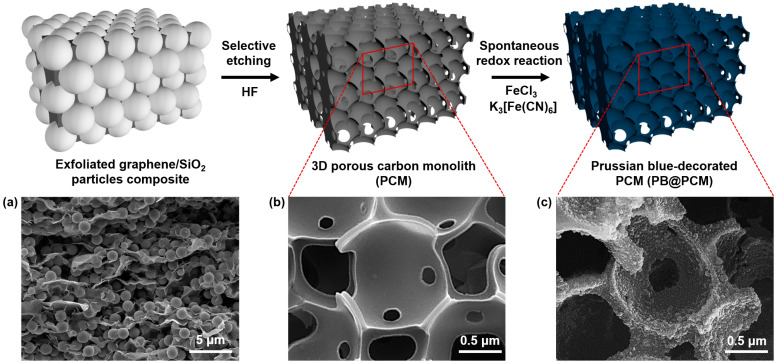
A schematic illustration for fabricating PCM through etching of the silica particle templates in EG/SiO_2_ composite and obtaining PB@PCM by a subsequent decoration of PB nanoparticles into the PCM. FE-SEM images of (**a**) EG/SiO_2_ particles composite, (**b**) PCM, and (**c**) PB@PCM.

The overall production procedure to obtain the PB@PCM is presented in Figure 1. In general, it is important to disperse the silica particles into the exfoliated graphene (EG) network. This was difficult to achieve because the EG-silica interactions were unfavorable and uniform mixing was almost impossible due to mild self-aggregation of the silica particles. To improve the EG-silica interactions, the surface of the silica particles was modified with amino silane coupling agent. The zeta potential value of silica particles increased from −4.03 to +6.17 mV after surface treatment (Supplementary Information, S1). Hence, the aggregation of silica particles could be suppressed and dispersion was promoted. The SEM image in Figure 1a shows no apparent trace of silica particle aggregation.

The particle locations were random and any regular texture was not observed. The external appearance of the EG/silica composite precursor was monolithic, because stacking of the EG sheets occurred spontaneously. In addition, this shape could be retained in the final product, PB@PCM.

Subsequently, the silica particles were removed by etching with HF and the PCM material was produced as shown in Figure 1b. A silica trace was clearly observed in the center of the FE-SEM image. Note that the PCM possessed an interconnected open pore structure and relatively small window pores. The formation of the window pores might be associated with local dewetting of the thin EG sheets, because the EG-silica particle interface was still less friendly after silane treatment. However, it could be inferred that the transport of radioactive ion species could be facilitated due to the presence of the window pores. The irregular pores might have originated from the inherent internal voids. It could be expected that the surface area increased after the silica etching.

**Figure 2:**
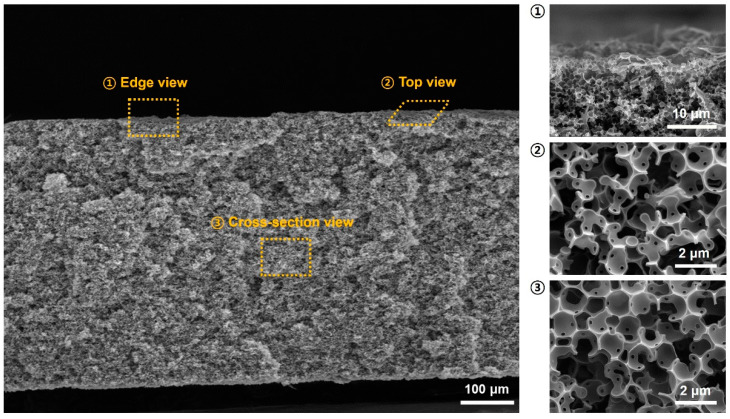
FE-SEM images of the 3D porous carbon monolith at different sections (edge, top, cross-section).

**Figure 3:**
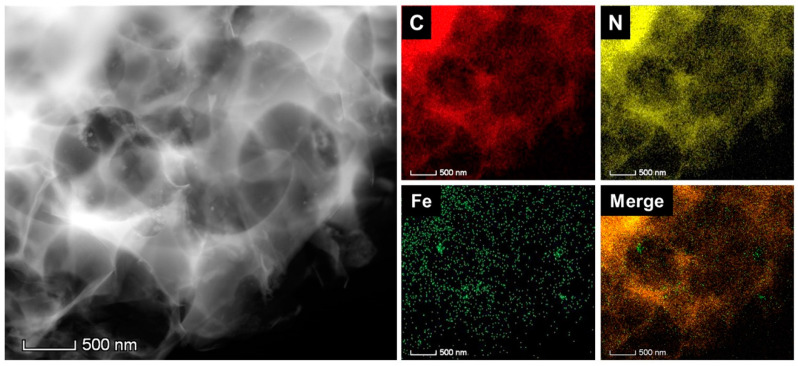
High-angle annular dark-field scanning TEM (HAADF-STEM) image and EDS elemental mapping images of PB@PCM for carbon ©, nitrogen (N), and iron (Fe). PB particles uniformly distributed over the whole PCM surface.

The PB decoration into the PCM materials led to the formation of the PB@PCM monolithic material, which would be used for removal of radioactive species. After the decorating with the PB nanoparticles, the rough surface of PCM material becomes smooth compared with that before the decoration, but there is no obvious difference in the structure or pore shape (Figure 1c). It was known that the PB decoration process was spontaneous and the average size of the PB nanoparticles was indiscernible [40,41,42,43]. The detailed internal structure of the PB@PCM is observed further in Figure 2 and Figure 3.

Figure 2 shows the macro and microscopic morphology of the PCM, which was uniform, and variation in appearance was negligible. This was because the components, EG and silica, in the precursor were homogeneous. It was difficult to observe any regular pore pattern and the pores were open and interconnected. The features shown in Figure 1b were observed analogously at the center and outer regions of the PCM monolith. It was concluded that the internal and external structure of the PCM was identical. This seemed desirable, because adsorption performance would be constant and independent.

### 2.2. Characterization of the Functionalized Porous Carbon Monolith

In this work, effective introduction of the PB nanoparticles and retention of porous structure were critical for PB@PCM production. A high-angle annular dark-field scanning TEM (HAADF-STEM) image of the PB@PCM indicates that the porous structure remained intact after PB addition (Figure 3). In addition, successful introduction of the PB nanoparticles was examined with EDS mapping analysis. It was verified that a coating layer of PB nanoparticles was introduced along the surface of the PCM. Several major elements such as C, N, O, and Fe were observed and traces of K and Cl also appeared (Supplementary Information, S2). The EDS mapping also provided the elemental compositions of the PB and PB@PCM. The Fe composition was high for PB nanoparticles, whereas C ratio increased for PB@PCM due to the presence of EG. The N and O compositions were almost constant regardless of the PB nanoparticle addition.

**Figure 4:**
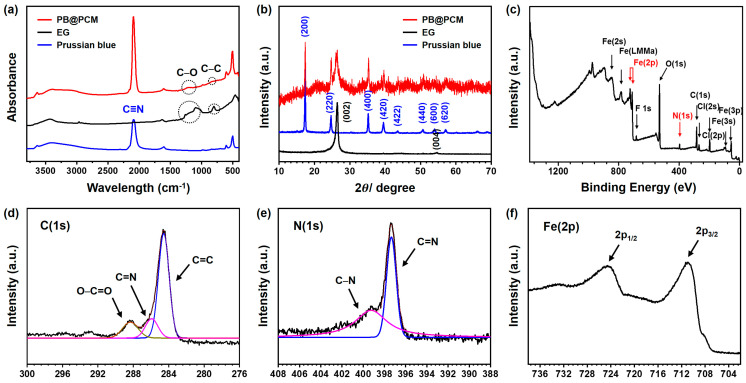
Characterization of Prussian blue, exfoliated graphene and PB@PCM with (**a**) FT-IR, (**b**) XRD. XPS survey spectra of PB@PCM (**c**) wide scan, (**d**) C(1s), (**e**) N(1s) and (**f**) Fe(2p).

The PB@PCM was further characterized using diverse instrumentations. The FT-IR spectrum of the EG showed weak peaks for C-O and C-C bending because there was a small amount of carbonyl groups on the EG (Figure 4a). A sharp peak for C≡N stretching appeared in the PB spectrum. All those features were seen in the PB@PCM spectrum, indicating that PB nanoparticles were successfully introduced to the surface of PCM. A similar trend was observed from the XRD profiles in Figure 4b. Peaks for highly crystalline PB nanoparticles were observed at 17(200), 25(220), 36(400), and 40(420) degrees, whereas that for crystalline EG was seen at 27(002) degrees [44].

Those representative diffraction peaks of PB and EG could also be assigned in the PB@PCM profile. The formation of a PB nanoparticle layer on the PCM was further confirmed by XPS. The survey spectrum of the PB@PCM in Figure 4c exhibited C(1s) (284.6 eV), O(1s) (531.2 eV), N(1s) (397.4 eV), and Fe(2p) (710.5 eV) peaks, indicating the substantial introduction of the PB nanoparticles into the PCM. The Gaussian fitting of the C(1s) peak in Figure 4d led to the occurrence of the three peaks: non-oxygenated C=C (288.3 eV, 69.41 at%), C=N (285.3 eV, 21.8 at%), and O-C=O (284.5 eV, 69.4 at%). The second one was highly associated with the presence of cyanide (C≡N) groups in the PB nanoparticles. The N(1s) peak was also fitted using the Gaussian method (Figure 4e) and showed the presence of two peaks: C-N (399.31 eV, 57.1 at%) and C=N (391.2 eV, 42.9 at%). In the fitted curve of the Fe(2p) in Figure 4f, two characteristic peaks of Fe (2p1/2) and Fe (2p3/2) were observed at 724.6 and 710.9 eV, respectively, both indicating the presence of α-FeOOH, whereas the peak at 708.2 eV was attributed to [Fe(CN)6] [45,46].

As an increase in surface area was important to achieve a high removal capacity, porous structure was introduced to the PB@PCM. N_2_ adsorption/desorption isotherms (BET) provided critical information regarding surface characteristics of the PB@PCM for practical application (Supplementary Information, S3). Note that the PB@PCM showed a stable adsorption behavior, indicating that it could adsorb a significantly higher amount of volume at relatively low pressure than the PCM. The structural parameters closely related to the surface properties were measured and are summarized in Table 1.

The specific area of the PB@PCM (128.76 m^2^/g) was approximately three times larger than that of PCM (43.49 m^2^/g), owing to the PB nanoparticle addition. Average pore diameter decreased from 0.35 to 0.27 μm after the PB addition. Even if the pore structure was irregular and wide open, the decrease in the average pore size was meaningful for adsorption capacity improvement. A significant change also occurred to other parameters except the total pore area after the PB introduction, suggesting that the change was attributed to the PB nanoparticle addition.

### 2.3. Adsorption/Decontamination Performances of the Functionalized Porous Carbon Monolith

**Figure 5:**
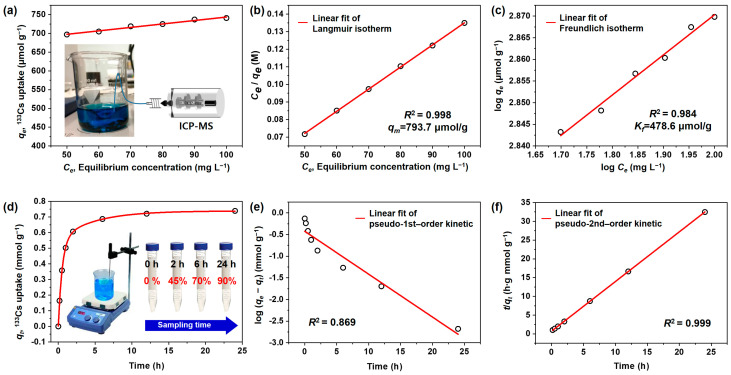
(**a**) ^133^Cs ion uptake behavior as a function of equilibrium ion concentration and adsorption isotherms for ^133^Cs ion uptake by the PB@PCM, linearly fitted to the (**b**) Langmuir and (**c**) Freundlich isotherm equation. (**d**) ^133^Cs ion uptake behavior as a function of contact time and adsorption isotherms for ^133^Cs ion uptake by the PB@PCM, linearly fitted to (**e**) pseudo-first-order and (**f**) pseudo-second-order kinetics.

To investigate the radioactive ion removal capacity of the PB@PCM, extensive adsorption tests were performed with the stable ^133^Cs isotope instead of the radioactive ^137^Cs. The ^133^Cs ion uptake behavior by the PB@PCM was monitored at pH 7.0 and 20 °C with varying ^133^Cs equilibrium concentration, as shown in Figure 5a.

In these experiments, the adsorption equilibrium was gradually established for 24 h after the addition of a designated amount of the PB@PCM to the test solutions. Accordingly, the adsorption capacity of the PB@PCM for ^133^Cs ion was measured after 24 h contact time. The ^133^Cs uptake by adsorption was calculated by the following equation:

(1) $$q_{e}=\frac{\left(C_{o}-C_{e}\right)V}{AW}$$

where *qe* is the equilibrium adsorption capacity (mmol/g) of adsorbent, *Co* and *Ce* the initial and equilibrium concentration (mg/L) of the ^133^Cs ion, V the total volume (L) of solution, A the atomic weight (g/mol) of ^133^Cs, and W is the weight (g) of the PB@PCM.

To analyze the adsorption behavior quantitatively, the equilibrium adsorption data was fitted linearly according to the Langmuir and Freundlich isotherms. Note that the Langmuir isotherm is dependent on an assumption that all the surface adsorption sites have identical affinity toward the adsorbate; hence, adsorption at one site does not affect the adsorption at an adjacent site [47]. In addition, each adsorbate molecule tends to occupy a single site; hence, monolayer formation is promoted on the adsorbent surface. The linear and nonlinear form of the Langmuir equations are as follows:

(2) $$\;\frac{C_{e}}{q_{e}}=\left(\frac{1}{q_{m}b}\right)+\left(\frac{1}{q_{m}}\right)C_{e}\;\left(\mathrm{linear}\;\mathrm{form}\right)\;\;\mathrm{or}\;\;q_{e}=\frac{q_{m}bC_{e}}{1+bC_{e}}\;\left(\mathrm{nonlinear}\;\mathrm{form}\right)$$

where *q_m_* is the maximum ^133^Cs uptake (mmol/g) and *b* the constant (L/mg) that refers to the bonding energy of adsorption related to free energy and net enthalpy. On the other hand, the Freundlich model includes the reversible adsorption at a heterogeneous surface; therefore, it is not restricted to monolayer formation [48]. The linear and nonlinear forms of the Freundlich adsorption isotherms are as follows:

(3) $$\log\;q_{e}=\log\;K_{f}+\frac{1}{n}\log\;C_{e}\;\left(\mathrm{linear}\;\mathrm{form}\right)\;\mathrm{or}\;q_{e}=K_{f}C_{e}^{1/n}\;\left(\mathrm{linear}\;\mathrm{form}\right)$$

where *K_f_* is the constant (mmol/g) related to the adsorption capacity of the adsorbent, and 1/*n* the intensity of the adsorption constant. Compared with the Langmuir isotherm, *K_f_* stands for analogous physical meaning to *q_m_*, even if those parameters are fundamentally different. Linear fitting was conducted according to the linear equation forms; consequently, the fitting results are shown in Figure 5b,c. Additional regression analysis was carried out by computer software (OriginPro 8.0, Originlab corporation, Northampton, MA, USA) to obtain the accurate parameters, and values are summarized in Table 2. It is clear that the fitting to the Langmuir isotherm showed a higher consistency with the adsorption data from the experiments with a regression coefficient of *R*^2^ = 0.999 and reasonable errors for parameters.

A supplementary parameter was suggested to further characterize the ^133^Cs adsorption behavior by the PB@PCM, which was separation factor (*R_L_*), a dimensionless constant [49]. *R_L_* value was calculated by the following equation:

(4) $$R_{L}=\frac{1}{1+bC_{0}}$$

where *b* and *C_o_* were the Langmuir constant and initial ^133^Cs concentration. The type of adsorption isotherm can be classified as unfavorable (*R_L_* > 1), linear (*R_L_* = 1), and favorable (*R_L_* = 0), depending on its value. The parameter obtained in this study was approximately 0.068, supporting the favorable adsorption of ^133^Cs on the PB@PCM. Therefore, it was concluded that the PB@PCM possessed desirable structural and surface features for ^133^Cs ion adsorption.

The adsorption behavior was also investigated as a function of contact time between ^133^Cs ions and PB@PCM. The curve in Figure 5d shows that the adsorption capacity increased rapidly during the initial 4 h and reached a maximum value (saturation) after 24 h. For quantitative analysis of the adsorption mechanism, the experimental data were fitted to the pseudo-first-order [50] and pseudo-second-order [51] kinetics and the results are exhibited in Figure 5e,f. The kinetics equations are as follows:

(5) $$\log\left(q_{e}-q_{t}\right)=\log q_{e}\;–\;\frac{K_{1}}{2.303}t\;\left(\mathrm{pseudo}-\mathrm{first}-\mathrm{order}\;\mathrm{equation}\right)$$

(6) $$\frac{t}{q_{t}}=\frac{1}{K_{2}{q_{e}}^{2}}+\frac{t}{q_{e}}\left(\mathrm{pseudo}\text{-}\mathrm{second}-\mathrm{order}\;\mathrm{equation}\right)$$

where *q_e_* and *q_t_* are the ^133^Cs uptake at equilibrium and time *t*, respectively, *K*_1_ the constant (1/min) for first-order adsorption, and *K*_2_ the rate constant (g/mmol·min) for second-order adsorption. The parameter values are summarized in Table 3 and it shows that the pseudo-second-order model described the adsorption mechanism better than the pseudo-first-order model with regression coefficients *R*^2^ = 0.999. The pseudo-second-order model is dependent on the assumption that the adsorption occurs on the adsorbent without interactions between adsorbates, and the desorption rate is negligible compared to the adsorption rate. It was inferred that the interactions between PB nanoparticles in PB@PCM and incoming radioactive ions could be facilitated due to the permanent/induced electrostatic forces between partially negative cyano groups (–C≡N) of PB nanoparticles and positive ^133^Cs ions. On the other hand, the interactions between the ions are supposed to be inconspicuous due to electrostatic repulsion.

To reveal the effectiveness of synergistic interaction between PB nanoparticles and PCM material, the ^133^Cs ion uptake test was performed using the PCM, bulk PB and PB@PCM (Supplementary Information, S4). It was important that the PB@PCM provided an improved uptake by adsorption compared with the PCM and bulk PB (A compilation of investigations carried out by using various PB-based adsorbents is also presented in Supplementary Information, S5 [52,53,54,55,56,57]). The maximum uptake value of PCM, bulk PB, and PB@PCM were ca. 0.0018, 0.0069, and 0.7225 mmol/g, respectively. It is noteworthy that the maximum ^133^Cs adsorption capacity of the PB@PCM is approximately 400 and 100 times higher than that of pristine PCM and bulk PB, respectively. This striking difference could be interpreted by the enhanced porosity and permeability of PCM and high surface area and large amount of PB nanoparticles in the synthesized PB@PCM, providing abundant active sites for ^133^Cs adsorption. Compared to the bulk PB, the PB@PCM containing both interconnected macroporous structure and nanometer-sized PB particles have enhanced adsorption properties due to increased mass transport through the material and maintenance of a specific surface area.

**Figure 6:**
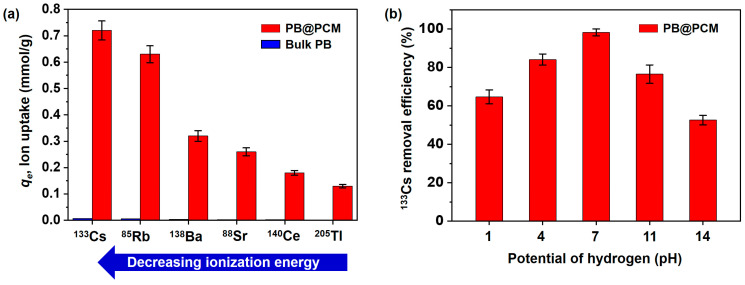
(**a**) Measurement of radioactive ion species uptake by the bulk PB and the PB@PCM to 6 radioactive ion species (^133^Cs, ^87^Rb, ^88^Sr, ^137^Ba, ^140^Ce, and ^205^Tl) at pH 7.0 and 20.0°C for 24 h. (**b**) The pH effect on the ^133^Cs removal efficiency of the PB@PCM at 20.0 °C for 24 h. Initial ^133^Cs ion concentration is 100 ppm.

It was also expected that the PB@PCM can adsorb various radioactive species; hence, uptake tests were performed at pH 7.0 and 20 °C with other radioactive ion species such as ^133^Cs, ^85^Rb, ^138^Ba, ^88^Sr, ^140^Ce, and ^205^Tl (Figure 6a). The adsorption capacity value varied from 0.72 to 0.13 mmol/g, with a decreasing order of ^133^Cs (0.72), ^85^Rb (0.63), ^138^Ba (0.32), ^88^Sr (0.26), ^140^Ce (0.18), and ^205^Tl (0.13). This trend might have originated from the degree of interaction between PB nanoparticles in PB@PCM and individual radioactive ion species. More positively charged ions could interact more favorably with the PB nanoparticles. It can be inferred that ^133^Cs might have a highly positive nature, because its electronegativity and ionization energy was lowest compared with other ion species (Supplementary Information, S6). However, the underlying correlation needs to be examined systematically in a separate research project.

In addition, the effect of the pH on the ^133^Cs adsorption capacity was also investigated (Figure 6b). Interestingly, the removal efficiency of the PB@PCM increased in acidic conditions and decreased in basic media, and the PB@PCM exhibited the highest uptake capacity at pH 7.0. This phenomenon can be ascribed to the competitive interaction between hydrogen (H+) and hydroxyl (OH−) ions during the adsorption process. In acidic condition (<pH 7.0), the H+ ion competes with the ^133^Cs ions towards the active sites of PB@PCM, which results in a relatively lower adsorption capacity. As the pH increased (≈pH 7.0), the decrease of the hydrogen ion concentration made it possible to increase the concentration of ^133^Cs ions to bind electrons, leading to a high adsorption uptake. However, when the basification was in progress (>pH 7.0), the stabilization attributed to the reaction between ^133^Cs ions and increased OH− ions occurred, and then the adsorption capacity decreased.

## 3. Materials and Methods

### 3.1. Materials

Tetraethyl orthosilicate (TEOS, 98%), 3-aminopropyltrimethoxysilane (APS), iron(III) chloride (FeCl_3_, 97%), potassium hexacyanoferrate(III) (K_3_Fe(CN)_6_, ≥99%), cesium, barium, strontium, and rubidium standard solution (1000 ppm) for inductively coupled plasma-mass spectrometer (ICP-MS) analyses were purchased from Sigma Aldrich (Milwaukee, WI, USA). Sodium chloride (NaCl), Ammonia solutions (NH_4_OH, 28.0~30.0%), hydrochloric acid (HCl), Ethanol (C_2_H_5_OH, ≥99%), and N-Methyl-2-pyrrolidone (NMP) were supplied from Samchun Chemical (Seoul, Korea). Hydrofluoric acid (HF, 40%) was purchased from J. T. Baker (Phillipsburg, NJ, USA). All chemicals were used as received without further purification. Electrochemically exfoliated graphene (EG) was produced according to the method reported previously [58].

### 3.2. Synthesis of Monodispersed Silica Microparticles

A certain amount of TEOS (36 g) was vigorously mixed with ethanol (200 mL) (Solution 1). An amount of ammonia solution (54 mL) and NaCl (0.1 g) was dissolved in a mixture of water (40 mL) and ethanol (190 mL) (Solution 2). Subsequently, solution 1 was slowly added to solution 2 at a speed of 1.2 μL/min using a micro syringe pump at 30 °C. Then the mixture solution was stirred for 1 h at 800 rpm. The produced microparticles were purified by centrifugation and washed with ethanol and distilled water 3 times. Finally, the obtained homogeneous silica particles were stored at room temperature.

### 3.3. Surface Modification of the Silica Particles

The obtained silica particles (1 g) were dispersed in ethanol (10 mL) by ultra-sonication for 10 min, resulting in a milky suspension. A small amount of glacial acetic acid (60 μL) was added to a mixture of ethanol (95 mL) and water (5 mL) to make the solution acidic (pH 5.0) and then APS (4.2 g, 19 mM) was added dropwise to the acidic solution. The resulting clear colorless solution was stirred (400 rpm) at RT for 15 min in order to form reactive silanol groups by hydrolysis.

### 3.4. Preparation of Porous Exfoliated Graphene Monolith

A certain amount of exfoliated graphene (EG, 240 mg) was dispersed in 20 mL of NMP and sonicated for 90 min to achieve a homogeneous mixing, which was critical for this experiment. Then, the silica particles were added to the EG solution, and the EG to silica weight ratio was 10:90. The obtained solution was filtered with a nylon membrane filter (47 mm in diameter, 0.2 μm pore size, Whatman) under vacuum suction to obtain an EG/silica composite precursor. The film type EG/silica composite was retrieved by peeling it off from the nylon membrane filter. A film type porous EG was obtained by removing the silica particles with HF solution (10%) for a few minutes. The PCM was dried in a vacuum oven at 120 °C overnight.

### 3.5. Fabrication of Prussian Blue Decorated Porous Carbon Monolith

The PB@PCM was manufactured by a redox reaction in an aqueous mixture solution of FeCl_3_, K_3_[Fe(CN)_6_], and PCM. Typically, PCM was soaked in 10 M FeCl_3_ solution for 15 min and the solution in sample was completely eliminated with a filter paper. Then, the PCM decorated with Fe^3+^ ions was dried in a convection oven at 80°C for at least 3 h. Then, the preprocessed PCM was immersed in a K_3_[Fe(CN)_6_] (0.64 g, 100 mL) solution and stirred at 55 °C for 12 h. The final PB@PCM product was obtained and washed with distilled water 3 times and dried under vacuum at 80°C overnight to complete evaporation of the solvent. The mass of the finally obtained PB@PCM sample was 2.4 g, which can be scaled up by increasing the amount of the EG/silica composite precursor. The bulk PB was fabricated by direct mixing of equimolar FeCl_3_ and K_4_[Fe(CN)_6_] solutions at room temperature for 6 h. The final products were washed with ethanol several times to remove residual reagent. Subsequently, the resulting bulk PB was dried under a vacuum oven and pressed into pellets.

### 3.6. Radioactive Material Extraction Test

The adsorption behavior of the PB@PCM for ^133^Cs ion was monitored as follows: the cesium standard solutions with varying concentration from 0.01 to 100 mg/L (ppm) were produced in 100 mL of distilled water. To control the pH, ammonia or hydrochloric acid solution was injected into the aqueous solutions. Then, 0.1 g of adsorbents (PB@PCM) was introduced into the solution and stirred at 300 rpm, which would be suitable for ^133^Cs ion adsorption. Adsorption experiments were carried out at 20 °C and pH 7.0, and the adsorption capacity for ^133^Cs ion was monitored from 0 min to 24 h. After adsorption, 3 mL of the solution was filtered through a syringe filter (PTFE, pore diameter 0.2 µm), and then an ICP-MS test was performed to determine the ^133^Cs ion concentration as a function of adsorption time. To obtain accurate results, the ^133^Cs ion concentration was measured three times and the average values were collected. The adsorption capacity to the other ions—rubidium, barium, strontium, cerium, and titanium—was also measured identically.

### 3.7. Characterization

The morphology was confirmed using a field emission scanning electron microscopy at 10 kV (Tescan Mira-3 FEG, Brno, Czech Republic) and transmission electron microscopy (FEI, Tecnai G2-20, Hillsboro, OR, USA). A particle size analyzer (Malvern, Zetasizer Nano-ZS, Worcestershire, United Kingdom) was used to determine the silica particle size and zeta potential. The FT-IR spectra were recorded on Alpha-P (Bruker, Ettlingen, Germany). The XRD patterns were collected using an Ultima IV with Cu Kα radiation (Rigaku, Tokyo, Japan). The BET analyses were performed with an ASAP 2020 (Micromeritics, Gwinnett County, GA, USA) using the N2 adsorption–desorption isotherms. The pore size and distribution in the porous carbon was analyzed with an AutoPore IV 9500 mercury porosimeter at room temperature (Micromeritics, Gwinnett County, GA, USA). The XPS data were obtained using an Axis Nova (KRATOS, Tokyo, Japan) with monochromatic Al-Kα X-ray source under 10-8 Torr vacuum analysis chamber. The ICP-MS was used to analyze and confirm the cesium concentration in solution (iCAP RQ, Thermo Fisher Scientific, Waltham, MA, USA).

## 4. Conclusions

In this study, convenient manufacture of monolithic porous carbon material embedded with Prussian blue particles was demonstrated and its radioactive substance removal performance was also examined using various radioactive ion species. The functional porous carbon was prepared by etching of silica template and subsequent Prussian blue nanoparticle decoration. The obtained porous carbon possessed an increased surface area compared with the precursor and the intermediate. In addition, its pore structure was open and interconnected with small window pores, which was advantageous for ion transport and movement. The radioactive substance removal was achieved by the incorporation of Prussian blue nanoparticles, which can adsorb radioactive ion species effectively. The ^133^Cs adsorption behavior could be described by both Langmuir and Freundlich isotherms, and fitting to the Langmuir isotherm provided a closer correlation with the experimental data. The porous carbon showed a better adsorption capacity than the precursor and an intermediate capacity for radioactive ion species such as ^133^Cs, ^87^Rb, ^88^Sr, ^137^Ba, ^140^Ce, and ^205^Tl. The maximum and minimum adsorption capacity values were obtained for ^133^Cs and ^205^Tl, respectively. As a porous carbon material was obtained by a relatively simple, cost-effective, and environmentally benign method, an interest in analogous functional porous materials for nuclear waste control is expected to increase significantly. Therefore, this research can offer important information for future relevant research and, moreover, development of advanced materials and devices.

## Figures and Tables

**Table 1:** Comparison of the parameters obtained from BET and Mercury Porosity Analysis.

Sample ^[a]^	Bulk Density	Total Pore Area	Specific Surface Area	Porosity	Avg. Pore Diameter
	(g/mL)	(m^2^/g)	(m^2^/g)	(%)	(μm)
PCM	0.41	21.76	43.49	78.04	0.35
PB@PCM	0.46	20.23	128.76	61.82	0.27

^[a]^ These values are estimated by using the BET and mercury porosimeter.

**Table 2:** The adsorption parameters obtained according to the Langmuir and Freundlich isotherms at room temperature for the adsorption of ^133^Cs to the PB@PCM.

	Langmuir ^[a]^			Freundlich ^[a]^		
	*q_m_* (μmol/g)	*b* (L/mg)	*R* ^2^	*K_f_* (μmol/g)	*n*	*R* ^2^
^133^Cs ^[b]^	793.651	0.136	0.999	478.631	10.782	0.984

^[a]^ *R*^2^ = regression coefficient. The *q_m_*, *b*, *K_f_*, *n*, values and the nonlinear regression correlations for Langmuir and Freundlich isotherms were measured by nonlinear regression analysis using OriginPro 8.0. ^[b]^ Three sets of valid data were collected, and the *C_e_* and *q_e_* values for ^133^Cs were calculated to be in the error range of ±5%.

**Table 3:** The kinetic parameters obtained using pseudo 1st-order and 2nd-order models at room temperature for the adsorption of ^133^Cs to the PB@PCM.

	Pseudo-First-Order ^[a]^			Pseudo-Second-Order ^[a]^		
	*K*_1_ (1/min)	*q_e_*_1_ (mmol/g)	*R* ^2^	*K*_2_ (g/mmol·min)	*q_e_*_2_ (mmol/g)	*R* ^2^
^133^Cs ^[b]^	0.228	0.424	0.936	2.395	0.755	0.999

^[a]^ *R*^2^ = regression coefficient. The *K*_1_, *K*_2_, *q_e_*_1_, *q_e_*_2_ values and the nonlinear regression correlations for pseudo-first-order and pseudo-second-order models were measured by nonlinear regression analysis using OriginPro 8.0. ^[b]^ Three sets of valid data were collected, and the *q_e_* and *q_t_* values for ^133^Cs were calculated to be in the error range of ±5%.

## Data Availability

Data sharing is not applicable to this article.

## Supplementary Materials

The following supporting information can be downloaded at: https://www.mdpi.com/article/10.3390/ijms23095116/s1.
