# Supplementary material for: Functionalization of Tailored Porous Carbon Monolith for Decontamination of Radioactive Substances
Source: Int J Mol Sci. 2022 May 4;23(9):5116. doi: 10.3390/ijms23095116 (PMC9105448; doi:10.3390/ijms23095116)
Supplement: Supplementary file 1 [file ijms-23-05116-s001.zip › ijms-1679317-supplementary.pdf]

Electronic Supplementary Information for

Functionalization of tailored porous carbon monolith for de-contamination of radioactive substances

Gyo Eun Gu [1§], Joonwon Bae [2§], Yeon Ju Kwon [1], Jea Uk Lee [3*] and Jin-Yong Hong [1*]

1 *Center for C1 Gas & Carbon Convergent Research, Korea Research Institute of Chemical Technology (KRICT), 141 Gajeong-ro, Yuseong-gu, Daejeon 34114, Republic of Korea; bodk7447@gmail.com (G.E.G.), kyj0905@krict.re.kr (Y.J.K)*

2 *Department of Applied Chemistry, Dongduk Women's University, Seoul 02748, Republic of Korea; red-sox7@dongduk.ac.kr (J.B.)*

3 *Department of Advanced Materials Engineering for Information and Electronics, Integrated Education Institute for Frontier Science & Technology (BK21 Four), Kyung Hee University, Gyeonggi-do, 17104, Republic of Korea*

* *Correspondence: leeju@khu.ac.kr; +82-31-201-3655 (J.U.L.), jyhong@krict.re.kr; +82-42-860-7591 (J.-Y.H.)*

§ *These authors contributed equally to this work.*

S1. Surface modification of silica particles

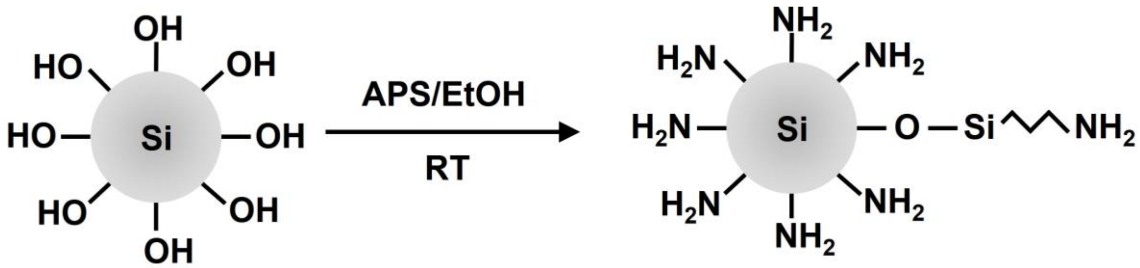

Figure S1. Schematic illustration for the surface modification mechanism during silanization reaction.

Table S1. The zeta potential values of silica particles before and after 3- aminopropyl-trimethoxysilane (APS) silane modification

Sample	Temperature (°C)	Zeta potential (mV)	Mobility (μmcm/Vs)	Conductivity (mS/cm)
Before modification	25	– 4.03	– 0.08145	0.0107
After modification	25	+ 6.17	0.1246	0.00178

[a] These values are acquired by zeta potential analyzer (Zetasizer Nano-ZS, Malvern)

S2. EDS elemental analysis data

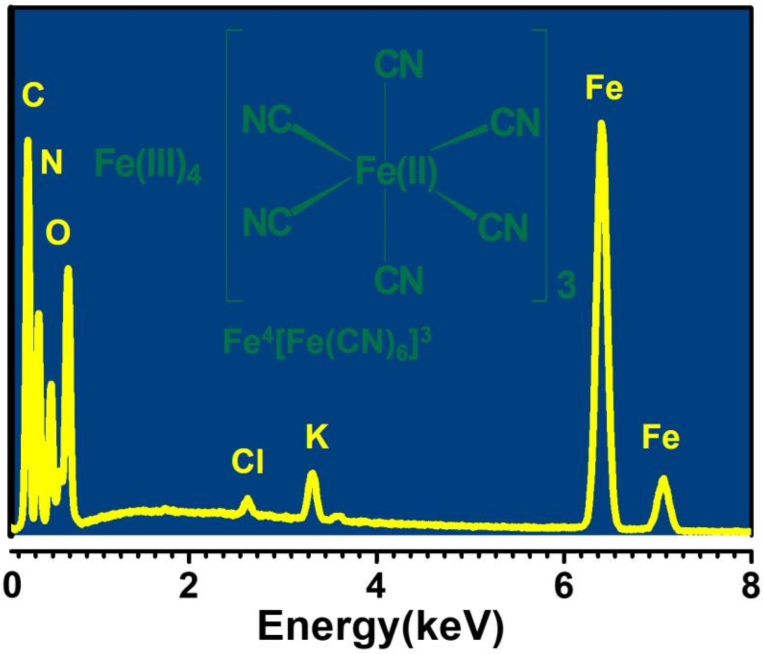

Figure S2. EDS spectrum of the PB@PCM

Table S2. Elemental analysis results of the PB and PB@PCM

Sample	Atomic ratio [a] (%)				
	**C**	**N**	**O**	**Fe**	**Cl**
PB	37.93	32.91	13.18	15.55	0.24
PB@PCM	41.15	35.26	13.31	9.6	0.13

S3. Pore characterizations

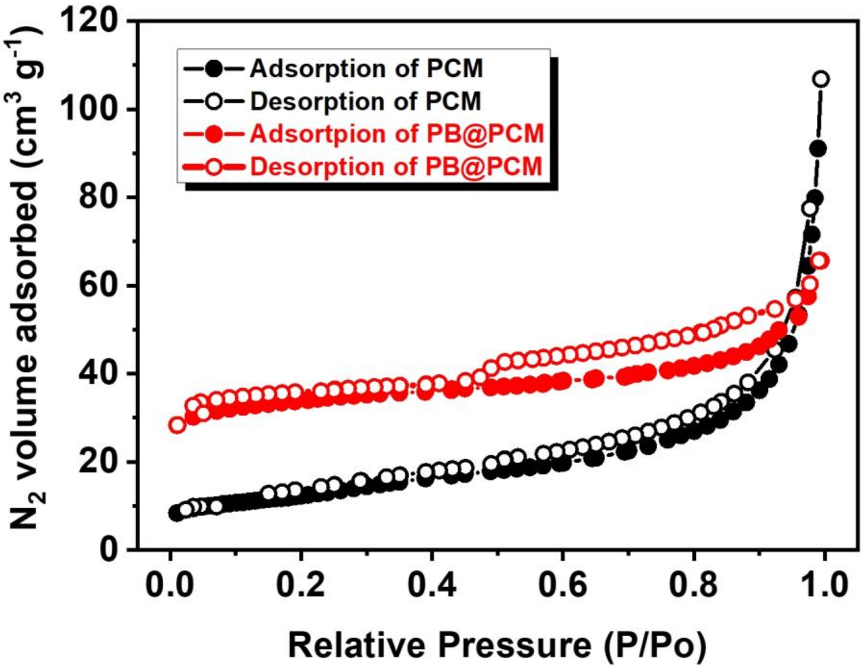

Figure S3. Pore characterization of the PCM and the PB@PCM. The BET surface areas and nitrogen adsorption–desorption isotherms were measured at 78 K using BELSORP-mini II analysis program.

S4. Comparison of adsorption capacities

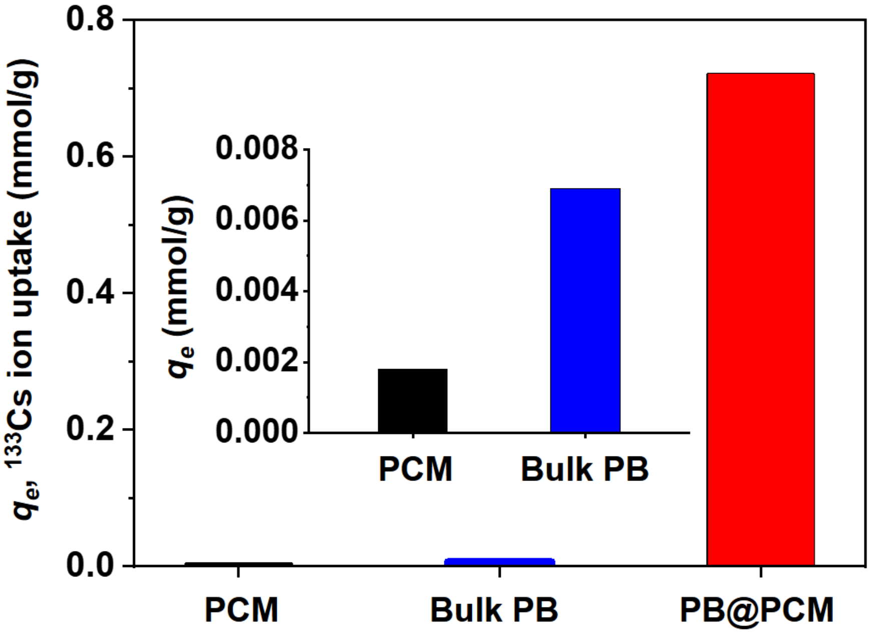

Figure S4. Comparison of adsorption capacities of the PCM (black color), bulk PB (blue color) and PB@PCM (red color) for removal of ^{133}Cs. Adsorption test were performed at pH 7.0 and 20.0°C for 24 h, and initial ^{133}Cs ion concentration is 100 ppm.

S5. A compilation of different investigations using various PB-based adsorbents

Table S3. Adsorption capacities of various PB-based adsorbents for cesium ion removal from solution

Adsorbent	Adsorption parameters	q_{max} (mg/g)	Ref.
COP-PAC-PB	Adsorbent amount: 0.01-5 g. Initial concentration: 50 mL of 10 ppm Cs solution. Contact time: 24 h. Temperature: RT	19.0	[1]
PB/Fe_3O_4/GO	Adsorbent amount: 50 mg. Initial concentration: 30 mL of 50-300 ppm Cs solution. Contact time: 24 h. Temperature: 25°C. pH: 7.0	55.6	[2]
PB-coated MNP	Adsorbent amount: 25 mg. Initial concentration: 4 mL of 50-2,780 ppm Cs solution. Contact time: 24 h.	96.0	[3]
PB/RGOF	Adsorbent amount: 10 mg. Initial concentration: 4 mL of 1-500 ppm Cs solution. Contact time: 12 h.	18.7	[4]
PB-MCs	Adsorbent amount: 30 mg. Initial concentration: 50 mL of 5-60 ppm Cs solution. Contact time: 4 h. Temperature: 20°C. pH: 5.5	4.8	[5]
PB/PAN	Initial concentration: 50 mL of 2-100 ppm Cs solution. Contact time: 24 h. Temperature: 25°C. pH: 7.0	93.4	[6]
PB@PCM	Adsorbent amount: 100 mg. Initial concentration: 100 mL of 100 ppm Cs solution. Contact time: 24 h. Temperature: 20°C. pH: 7.0	98.5	This work

S6. Atomic properties of radionuclides

Table S4. Atomic properties of various radioactive materials

**Element**[a]	**Electro-negativity**	**Ionization energy (kJ/mol)**			**Atomic radious**	**Covalent radius**
		**1st**	**2nd**	**3rd**	**(pm)**	**(pm)**
Cesium (Cs)	0.79	375.7	2234.3	3400.0	265	244 ± 11
Rubidium (Rb)	0.82	403.0	2632.1	3859.4	248	220 ± 9
Barium (Ba)	0.89	502.9	965.2	3600.0	222	215 ± 11
Strontium (Sr)	0.95	549.5	1064.2	4138.0	215	195 ± 10
Cerium (Ce)	1.12	534.4	1050.0	1949.0	181	204 ± 9
Thallium (Tl)	1.62	589.4	1971.0	2878.0	170	145 ± 7

[a] Several elements were selected as representative radionuclides.

References

1. Kim, B., Oh, D., Kang, S., Kim, Y., Kim, S., Chung, Y., Seo, Y., Hwang, Y., 2019. Reformation of the surface of powdered activated carbon (PAC) using covalent organic polymers (COPs) and synthesis of a Prussian blue impregnated adsorbent for the decontamination of radioactive cesium. J. Alloys. Compd. 785, 46–52.
2. Yang, H., Sun, L., Zhai, J., Li, H., Zhao, Y., Yu, H., 2014. In situ controllable synthesis of magnetic Prussian blue/graphene oxide nanocomposites for removal of radioactive cesium in water. J. Mater. Chem. A 2, 326–332.
3. Thammawong, C., Opaprakasit, P., Tangboriboonrat, P., Sreearunothai, P., 2013. Prussian blue-coated magnetic nanoparticles for removal of cesium from contaminated environment. J. Nanopart. Res. 15, 1689–1699.
4. Jang, S.C., Haldorai, Y., Lee, G.W., Hwang, S.K., Han, Y.K., Roh, C., Huh, Y.S., 2015. Porous three-dimensional graphene foam/Prussian blue composite for efficient removal of radioactive (137)Cs. Sci. Rep. 5, 17510.
5. Feng, S., Li, X., Ma, F., Liu, R., Fu, G., Xing, S., Yue, X., 2016. Prussian blue functionalized microcapsules for effective removal of cesium in a water environment. RSC Adv. 6, 34399–34410.
6. Jia, Z., Cheng, X., Guo, Y., Tu, L., 2017. In-situ preparation of iron(III) hexacyanoferrate nano-layer on polyacrylonitrile membranes for cesium adsorption from aqueous solutions. Chem. Eng. J. 325, 513–520
